# Chemical composition of *Callistemon subulatus* essential oils and the protective effects against cyclophosphamide-induced lung toxicity: GC/MS metabolic profiling with *in vivo* and *in silico* experimental studies

**DOI:** 10.3389/fphar.2026.1795229

**Published:** 2026-04-14

**Authors:** Omyma Rabie, Heba A. S. El-Nashar, Mina Y. George, Eman F. Khaleel, Rehab Mustafa Badi, Mahmoud A. EL Hassab, Wagdy Eldehna, Nada M. Mostafa

**Affiliations:** 1 Department of Pharmacognosy, Faculty of Pharmacy, Ain Shams University, Cairo, Egypt; 2 Department of Pharmacology and Toxicology, Faculty of Pharmacy, Ain Shams University, Cairo, Egypt; 3 Biology Department, School of Pharmacy, Newgiza University, Giza, Egypt; 4 Department of Medical Physiology, College of Medicine, King Khalid University, Asir, Saudi Arabia; 5 Department of Medicinal Chemistry, Faculty of Pharmacy, King Salaman International University (KSIU), South Sinai, Egypt; 6 Department of Pharmaceutical Chemistry, Faculty of Pharmacy, Kafrelsheikh University, Kafrelsheikh, Egypt

**Keywords:** *Callistemon subulatus*, cyclophosphamide, docking, eucalyptol, oxidative stress

## Abstract

*Callistemon* is a genus of aromatic plants indigenous to Australia and widely spread for ornamental purposes. In this study, we aimed to investigate the chemical composition of essential oils isolated from the leaves, flowers, stems, and fruits of *Callistemon subulatus* using gas chromatography–mass spectrometry (GC/MS) analysis, alongside the assessment of the protective effects of the leaf oil against cyclophosphamide (CY)-induced lung toxicity in rats. The leaf oil was rich in oxygenated monoterpenes (51.15%), while the flower oil contained a higher proportion of monoterpene hydrocarbons (72.06%). The stem and fruit oils were predominantly composed of hydrocarbons, accounting for 52.79% and 62.52%, respectively. Interestingly, *C. subulatus* leaf oil (CLO) showed the highest yield with the highest proportion of eucalyptol (44.36%), a reported anti-inflammatory monoterpene. Thirty rats were divided into five groups and treated for 10 days as follows: group I is a vehicle group, group II was administered CY (200 mg/kg, *i. p.*), groups III and IV received oral CLO (50 and 100 mg/kg, respectively) in addition to CY (200 mg/kg, *i. p.*), and group V received only CLO (100 mg/kg, oral). It was found that CLO inhibited the lipid peroxidation level and augmented catalase and glutathione levels. Furthermore, it significantly reduced the overtranscription of *α*-smooth muscle actin (*α-*SMA) by preventing fibroblastic cell differentiation into myofibroblasts. The histological appearance of CY-induced lung tissue revealed congestion, epithelial hyperplasia, and inflammatory cell aggregations. However, CLO considerably alleviated these manifestations. The findings demonstrated the preventive function of CLO against the harmful effects of CY on rat lungs. Therefore, *C. subulatus* oil might be a promising natural candidate for protecting the lung from CY-induced pneumotoxic manifestations.

## Graphical Abstract

**Figure:**
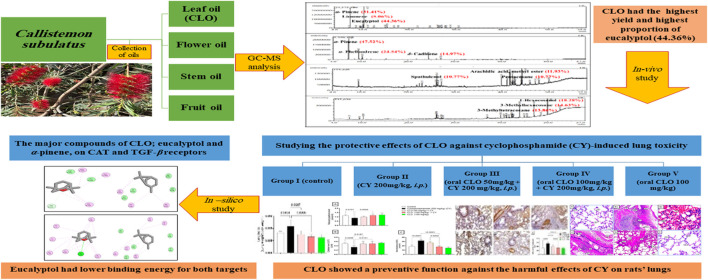

## 1 Introduction

Cyclophosphamide (CY) is a commonly used chemotherapeutic alkylating agent for the treatment of breast cancer, non-Hodgkin’s lymphoma, multiple myeloma, sarcoma, and leukemia (Singh et al., 2023). It is also used as an immunosuppressive in organ transplantation and autoimmune conditions such as rheumatoid arthritis, systemic lupus erythematosus, lupus nephritis, and multiple sclerosis (Bakr et al., 2023). It is a pro-drug that requires metabolism in the liver by cytochrome P450 enzymes to release its two main cytotoxic metabolites, acrolein and phosphoramide mustard (Kachur et al., 2023). The latter is an active form that forms covalent bonds with alkyl groups in DNA, thereby obstructing DNA replication and inducing apoptosis. Unfortunately, side effects have been reported with CY, and the metabolite acrolein is thought to be responsible for these adverse effects (Barberino et al., 2023). These include pneumotoxicity, cardiotoxicity, hepatotoxicity, nephrotoxicity, an increased risk of hemorrhagic cystitis, and myelosuppression leading to septic shock (Brummaier et al., 2013).

The pulmonary toxicity caused by CY was clinically well documented, dose-related, and manifested as either early-onset interstitial pneumonitis that presents as cough, hyperthermia, and dyspnea or late-onset pulmonary fibrosis with pleural thickening and non-productive cough that progresses to severe pulmonary insufficiency (Bartl et al., 2023). The explanation for this pneumotoxicity is that CY is partly metabolized in the lungs, which may result in the generation of highly cytotoxic reactive oxygen species (ROS) that cause pulmonary damage (Conte et al., 2022). Consequently, CY administration was found to be linked to the depletion of reduced glutathione (GSH) and catalase (CAT), along with enhanced lipid peroxidation, as evidenced by elevated malondialdehyde (MDA) levels (Cengiz et al., 2020). When this redox imbalance exceeds a certain level, CY recruits and infiltrates inflammatory cells, such as macrophages, neutrophils, and monocytes, which, in turn, causes a severe inflammatory reaction (El-Kholy et al., 2017). Activation of these inflammatory cells leads to increased fibroblast differentiation into myofibroblasts, thereby increasing the transcription of the profibrotic *α*-SMA gene (Yu et al., 2022). In pulmonary fibrosis, *α-*SMA-expressing myofibroblasts accumulate and produce collagen and extracellular matrix proteins in alveolar structures.

Atypical cells in the alveolar and bronchiolar epithelium, hyperplasia of pneumocytes, alveolar edema and fibrosis, and lymphocytic interstitial infiltration were among the prevalent clinical histopathological findings in CY-induced pneumotoxicity (Mohamed et al., 2020).

Numerous studies have demonstrated that natural agents function as lung protectants; when given in conjunction with CY, they reduced oxidative stress, decreased the secretion of pro-inflammatory cytokines, and restored normal lung architecture, thereby mitigating CY-induced pulmonary damage. These include allicin (Ashry et al., 2013), quercetin (Şengül et al., 2017), curcumin (Saghir et al., 2020), raspberry ketones (Mohamed et al., 2020), lutein, *β*-cryptoxanthin (Badawi, 2022), and the extracts of *Origanum vulgare* (Shokrzadeh et al., 2014a), *Zataria multiflora* (Habibi et al., 2020), and *Saussurea costus* roots (Attallah et al., 2023). Therefore, natural antioxidant sources can serve as CY-adjunctive therapy and can lessen the medication’s negative effects without compromising its efficacy (Younis et al., 2023; Imran et al., 2023; El-Nashar et al., 2023).

The *Callistemon* genus comprises evergreen woody shrubs or small trees with fragrant, lanceolate leaves (Sharma et al., 2021). It belongs to the family Myrtaceae and has 37 species. This genus is indigenous to Australia, where it has been used as an antimicrobial, anthelmintic, and insecticide agent, as well as a hot tea and traditional herbal medicine for bronchitis, gastroenteritis, and respiratory infections (Rathore and Rai, 2022). Since *Callistemon* is an aromatic genus, every portion of the plant contains volatile oil, and the main monoterpenes in most species include 1,8 cineole (eucalyptol), *α*-terpineol, and *α*-pinene (Gad et al., 2019). Various biological activities have been reported for *Callistemon* essential oils, such as antioxidant and free radical scavenging, antitumor, antimicrobial, anticonvulsant, anti-aging, and insecticidal effects (Abdelmalek et al., 2021; Bhandari et al., 2021).

We aimed to investigate the chemical composition of the essential oils from different organs of *C. subulatus,* including leaves, flowers, stems, and fruits, which were harvested simultaneously in late spring. In addition, we aimed to assess the potential protective effects of leaf oil against CY-induced pneumotoxicity in rats using biochemical and histological examinations of lung tissues.

## 2 Materials and methods

### 2.1 Drugs and chemicals

Cyclophosphamide was purchased from Baxter Oncology GmbH, Germany, and the anti-*α*-smooth muscle actin (Catalog No. ab7817) antibody was purchased from Abcam, United States. All other chemicals and buffers were of the highest commercially available purity grade.

### 2.2 Plant material

The four parts of *C. subulatus* (leaves, flowers, stems, and fruits) were harvested in the spring of 2021 from El Orman Garden, Giza, Egypt. Mrs. Treize Labib, a taxonomist at the Egyptian Ministry of Agriculture, kindly identified the plant. The voucher specimen (PHG-P-CS-416) was placed in the Herbarium of the Pharmacognosy Department, Faculty of Pharmacy, Ain Shams University.

### 2.3 Isolation of essential oils

The fresh parts were divided into four organs: leaves (550 gm), flowers (100 gm), stems (450 gm), and fruits (capsules) (450 gm). Each part was individually hydrodistilled using a Clevenger apparatus for 4 h. Each essential oil was gathered and stored in a firmly sealed vial before being chilled at 4 °C for further analysis.

### 2.4 Gas chromatography–mass spectrometry analysis

Gas chromatography–mass spectrometry (GC/MS) analysis was performed using a Shimadzu GC/MS-QP2010 apparatus (Japan) equipped with a fused-bonded RTX-5 column (Restek, United States) (30 m × 0.25 mm i. d. × 0.25 µm film thickness). The flow rate of helium, used as the carrier gas, was set to 1.0 mL/min. The oven temperature was held at 80 °C for 2 min, increasing to 300 °C at a rate of 5.0 °C/min, and then held for a final 5 min. Approximately 0.2 µL of diluted oil samples were automatically injected at a split ratio of 1:15 and an injector temperature of 250 °C. The electron ionization (EI) mode (70 eV), filament emission current (60 mA), and ion source temperature (200 °C) were used to record mass spectra with a 20–500 m/z spectral range. The obtained retention indices (RIs) were compared to those in the online NIST library and the literature to determine the compositional identity (El-Kersh et al., 2022).

### 2.5 Molecular docking study

The three-dimensional X-ray structures of human CAT and the transforming growth factor-*β* (TGF-*β*) receptor were retrieved from the Protein Data Bank under the IDs 8hid and 6b8y, respectively. Molecular docking was performed using AutoDock Vina and MGL Tools (Trott and Olson, 2010; El Ha et al., 2025). Two predominant constituents of *Callistemon* oil, eucalyptol and *α*-pinene, were selected for the docking analysis. Both receptors and ligands were converted into the pdbqt format using MGL tools, as required for AutoDock Vina. The binding sites of the targets were identified based on their co-crystallized ligands. Docking outcomes were then analyzed using the Discovery Studio Visualizer, which was also used to construct 2D interaction diagrams.

### 2.6 Animals

Male Wistar rats, 10 weeks of age and weighing 200–220 g, were purchased from the National Research Center, Cairo, Egypt. The rats were allowed a 2-week acclimatization period. They were supplied with food and water *ad libitum*. The study protocol was conducted according to the institutional and ethical guidelines of the care and use of laboratory animals, complied with the ARRIVE guidelines, and was approved by the ethical committee of the Faculty of Pharmacy, Ain Shams University (Egypt) (ENREC-ASU-number 133). Rats were subjected to alternative 12 h light and dark cycles and housed in an air-conditioned atmosphere (25 °C).

### 2.7 Experimental design

Thirty male rats were randomly divided into five groups of six rats each and received the following treatments for 10 days: Group I (negative control group) received *i. p.* 0.9% sodium chloride daily; Group II received a single dose of CY (200 mg/kg, i. p.) on day 7; Groups III and IV were administered CLO at doses of 50 and 100 mg/kg, respectively, via oral gavage for 10 consecutive days. The CLO was suspended in distilled water. Additionally, they received a single dose of CY (200 mg/kg, i. p.) on day 7; Group V received oral CLO only at a dose of 100 mg/kg for 10 consecutive days.

Previous research has shown that eucalyptus oil and spearmint oil had a protective effect against pulmonary destruction and lung injury in COPD rats at levels of 30–300 mg/kg. To be more particular about *Callistemon* oils, *C. lanceolatus* leaf oil at 50 and 100 mg/kg was tested for anti-inflammatory efficacy in rat models (Wang et al., 2017; Zhao et al., 2008; Sudhakar et al., 2004). Therefore, these doses (50 and 100 mg/kg) were chosen to assess the pharmacological activity of CLO on rat lung tissues.

After the experiment period, animals were sacrificed, and lung tissues from all groups were collected. Samples were fixed in 10% buffered formalin and added to paraffin blocks for histological examination and immunohistochemical detection. Other specimens were homogenized at 1:10 (w:v) in potassium phosphate buffer (pH 7.5) for further biochemical analysis.

### 2.8 Assessment of the lung/bodyweight index

Lung indices for different study groups were calculated as lung weight/body weight (Habib et al., 2023).

### 2.9 Assessment of oxidative stress markers

GSH levels were assessed using kits from Biodiagnostics, Giza, Egypt, following a previously reported method (Beutler et al., 1963). The method is based on the reduction of 5,5′-dithiobis-(2-nitrobenzoic acid) (DTNB) by GSH to form a yellow-colored chromogen, which is measured spectrophotometrically at 412 nm. Results were expressed as mmol/mL. The levels of glutathione were determined using a calibration curve generated from concentrations of reduced glutathione (0.1–1 mmol/mL).

Moreover, the antioxidant CAT enzyme level was determined using kits purchased from Biodiagnostics, Giza, Egypt. The catalase level was colorimetrically measured according to previous methodology (Aebi, 1984). The assay is based on the ability of catalase to decompose hydrogen peroxide (H_2_O_2_). The decrease in hydrogen peroxide concentration was measured spectrophotometrically at 240 nm, and catalase activity was expressed in U/mL. Catalase activity was determined by measuring the rate of hydrogen peroxide decomposition and expressed in U/mL according to the assay protocol.

In addition, lipid peroxidation levels were detected by measuring thiobarbituric acid (TBA)-reactive substances (TBARSs) such as MDA (Satoh, 1978). The assay is based on the reaction of MDA with TBA under high temperature and acidic conditions to form a TBARS, producing a pink chromogen that was measured spectrophotometrically at 534 nm. Results were expressed as nmol MDA per mL. Malondialdehyde levels were calculated using a standard curve prepared with 1,1,3,3-tetramethoxypropane (0.3–20 nmol/mL).

### 2.10 Immunohistochemistry

Immunohistochemical detection of *α*-SMA using its primary antibody was performed to assess the expression level. Counterstaining was performed using hematoxylin. Slides were visualized under a microscope (Olympus BX-50 Olympus Corporation, Tokyo, Japan). Image analysis was performed using ImageJ software, 1.48a, NIH, United States (Awad et al., 2023).

### 2.11 Histological examination

After fixation, washing, and preparation of paraffin blocks, lung tissue blocks were cut using a slide microtome (4 µm thickness) and deparaffinized. Afterward, hematoxylin and eosin staining was carried out. Then, visualization was performed using an Olympus microscope (BX-50 Olympus Corporation, Tokyo, Japan) (George et al., 2021).

### 2.12 Statistical analysis

Data are presented as mean ± SD. Multiple comparisons were performed using one-way ANOVA followed by Tukey’s post-hoc test. A probability level of 0.05 was used as the criterion for significance. All statistical analyses and graph plotting were carried out using GraphPad Prism software version 9 (GraphPad Software, Inc., La Jolla, CA, United States).

## 3 Results

### 3.1 Chemical analysis of the essential oils isolated from different organs of *C. subulatus*

**FIGURE 1:**
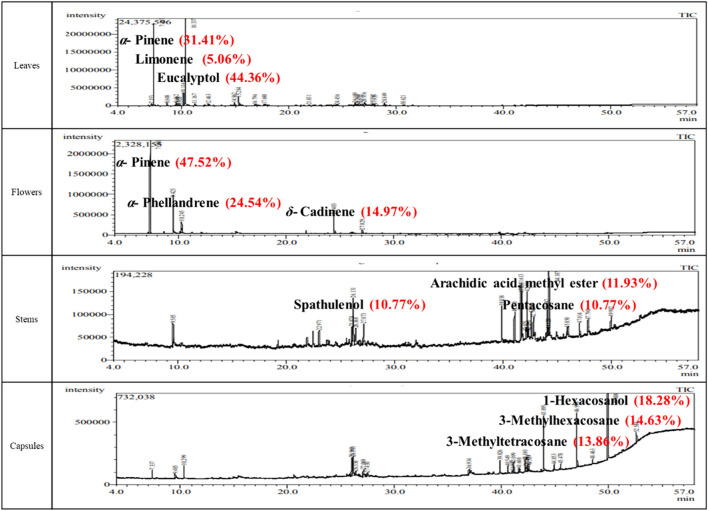
GC chromatograms of *C. subulatus* essential oils showing their compositional variation.

GC–MS analysis was performed to compare the volatile constituents of leaves, stems, fruits, and flowers of *C. subulatus*. A total of 51 compounds were identified, and it was demonstrated that fruit oil had 25 compounds, leaf oil included 24, stem oil contained 22, and flower oil had 5 compounds. The identified 51 compounds, along with the differences in yield and chemical composition among the different plant organs, are shown in Figure 1 and Table 1. Among the tested oils, the leaf oil showed the highest yield with a predominance of eucalyptol (44.36%), *α*-pinene (31.4%), limonene (5.06%), and *α*-terpineol (3.62%). Meanwhile, *α*-pinene (47.52%), *α*-phellandrene (24.54%), and *δ*-cadinene (14.97%) were predominant in the flower oil. For the first time, the stem and fruit oils of *C. subulatus* have been chemically investigated. The most prevalent component of the stem oil is arachidic acid methyl ester (11.93%), followed by spathulenol (10.77%), pentacosane (10.77%), and 2-methyltricosane (9.24%). However, the most abundant constituents of fruit oil were 1-hexacosanol (18.28%), 3-methylhexacosane (14.63%), 3-methyltetracosane (13.86%), and spathulenol (8.18%).

**TABLE 1:** GC/MS analysis of *C. subulatus* leaf, flower, stem, and capsule oils in spring.

No.	Compound name	Class	Retention time Rt	Molecular formula	RI_exp_	RI_lit_	Relative abundance %			
							Leaf	Flower	Stem	Capsule
1	*α-*Thujene	MT	7.155	C_10_H_16_	908	906	0.64	-	-	-
2	*α*-Pinene	MT	7.335	C_10_H_16_	914	917	31.41	**47.52**	-	1.51
3	(−)-β-Pinene	MT	8.610	C_10_H_16_	962	963	0.72	-	-	-
4	*α*-Phellandrene	MT	9.460	C_10_H_16_	992	994	1.03	24.54	3.10	1.13
5	2-Carene	MT	9.635	C_10_H_16_	999	1000	0.10	-	-	-
6	3-Carene	MT	9.755	C_10_H_16_	1003	1004	0.17	-	-	-
7	Limonene	MT	10.115	C_10_H_16_	1016	1018	5.06	-	-	-
8	Eucalyptol	OM	10.245	C_10_H_18_O	1022	1022	**44.36**	9.76	-	2.99
9	γ-Terpinene	MT	11.165	C_10_H_16_	1049	1049	0.76	-	-	-
10	*α*-Terpinolene	OM	12.465	C_10_H_16_	1090	1085	0.74	-	-	-
11	Terpinen-4-ol	OM	14.860	C_10_H_18_O	1168	1168	1.27	-	-	-
12	*α-*Terpineol	OM	15.285	C_10_H_18_O	1181	1181	3.62	-	-	-
13	β-Citral	OM	16.795	C_10_H_16_O	1232	1232	0.63	-	-	-
14	α-Citral	OM	17.680	C_10_H_16_O	1263	1267	1.27	-	-	-
15	Caryophyllene	ST	21.830	C_15_H_24_	1409	1409	0.27	-	-	-
16	Alloaromadendrene	ST	22.970	C_15_H_24_	1454	1453	-	-	2.05	-
17	*δ*-Cadinene	ST	24.455	C_15_H_24_	1512	1515	0.56	14.97	-	-
18	Ledol	OS	25.980	C_15_H_24_O	1572	1572	-	​	3.53	6.37
19	Spathulenol	OS	26.130	C_15_H_24_O	1577	1577	1.39	-	10.77	8.18
20	Viridiflorol	OS	26.340	C_15_H_26_O	1587	1587	0.61	-	4.56	2.29
21	Guaiol	OS	26.610	C_15_H_26_O	1596	1600	0.44	-	-	-
22	Caryophyllene oxide	OS	27.030	C_15_H_24_O	1616	1615	2.34	3.22	3.72	1.59
23	*α*-Acorenol	OS	27.430	C_15_H_24_O	1631	1631	-	-	-	1.09
24	Guaia-3,9-diene-11-ol	OS	27.790	C_15_H_24_O	1647	1649	0.57	-	-	-
25	Neointermedeol	OS	27.935	C_15_H_26_O	1653	1656	0.39	-	-	-
26	Germacrone	OS	28.850	C_15_H_22_O	1693	1696	1.40	-	-	-
27	3-Methylheptadecane	MA	30.620	C_18_H_38_	1770	1772	0.27	-	-	-
28	3-Methyleicosane	MA	36.935	C_21_H_44_	2079	2072	-	-	-	1.53
29	Nonadecanoic acid methyl ester	FE	39.840	C_20_H_40_O_2_	2238	2230	-	-	5.57	4.18
30	3-Methyldocosane	MA	40.550	C_23_H_48_	2278	2275	-	-	-	2.05
31	Tricosane	A	41.000	C_23_H_48_	2303	2300	-	-	4.76	0.48
32	2-Heneicosanone	Other	41.100	C_21_H_42_O	2308	2309	-	-	-	2.82
33	Arachidic acid methyl ester	FE	41.615	C_21_H_42_O_2_	2336	2333	-	-	**11.93**	1.27
34	5-Methyltricosane	MA	41.655	C_24_H_50_	2338	2347	-	-	5.39	-
35	4-Methyltricosane	MA	42.025	C_24_H_50_	2359	2353	-	-	3.48	-
36	2-Methyltricosane	MA	42.190	C_24_H_50_	2368	2365	-	-	9.24	3.57
37	3-Methyltricosane	MA	42.290	C_24_H_50_	2372	2371	-	-	1.05	1.23
38	Arachidic acid	F	42.410	C_20_H_40_O_2_	2380	2380	-	-	-	2.20
39	Tetracos-1-ene	AE	42.530	C_24_H_48_	2387	2396	-	-	4.17	1.69
40	Tetracosane	A	42.745	C_24_H_50_	2398	2400	-	-	3.83	-
41	3-Methyltetracosane	MA	43.895	C_25_H_52_	2472	2473	-	-	4.59	13.86
42	Pentacos-1-ene	AE	44.120	C_25_H_50_	2486	2486	-	-	0.89	-
43	Pentacosane	A	44.185	C_25_H_52_	2490	2500	-	-	10.77	-
44	Behenic acid methyl ester	FE	44.855	C_23_H_46_O_2_	2533	2531	-	-	-	1.87
45	3-Methylpentacosane	MA	45.480	C_26_H_54_	2574	2573	-	-	-	1.17
46	Hexacosane	A	45.850	C_26_H_54_	2597	2600	-	-	0.94	-
47	3-Methylhexacosane	MA	47.015	C_27_H_56_	2671	2673	-	-	1.71	14.63
48	Tetracosanoic acid, methyl ester	FE	47.790	C_25_H_50_O_2_	2722	2725	-	-	1.97	-
49	2-Methylheptacosane	MA	48.465	C_28_H_58_	2765	2761	-	-	-	1.21
50	1-Hexacosanol	Other	49.900	C_26_H_54_O	2857	2852	-	-	1.97	**18.28**
51	Heptacosanoic acid methyl ester	FE	52.585	C_28_H_56_O_2_	3030	3027	-	-	-	2.80
​	Hydrocarbon monoterpene (MT) %	​	​	​	​	​	40.66	72.06	3.10	2.64
​	Oxygenated monoterpene (OM) %	​	​	​	​	​	51.15	9.76	-	2.99
​	Hydrocarbon sesquiterpene (ST) %	​	​	​	​	​	0.83	14.97	2.05	-
​	Oxygenated sesquiterpene (OS) %	​	​	​	​	​	7.14	3.22	22.58	19.52
​	Fatty acid (F) %	​	​	​	​	​	-	-	-	2.20
​	Fatty acid ester (FE) %	​	​	​	​	​	-	-	19.47	10.12
​	Hydrocarbons total %	​	​	​	​	​	0.27	-	52.79	62.52
	Alkanes (A) %	​	​	​	​	​	-	-	20.3	0.48
	Monomethyl alkanes (MA) %	​	​	​	​	​	0.27	-	25.46	39.25
	Alkenes (AE) %	​	​	​	​	​	-	-	5.06	1.69
	Others %	​	​	​	​	​	-	-	1.97	21.1
​	Total identified %	​	​	​	​	​	100.02	100.01	99.99	99.99
​	Yield of oil (mL/100 g)	​	​	​	​	​	1.56	0.25	0.36	0.59

Bold values indicate the main component were found in each oil.

**FIGURE 2:**
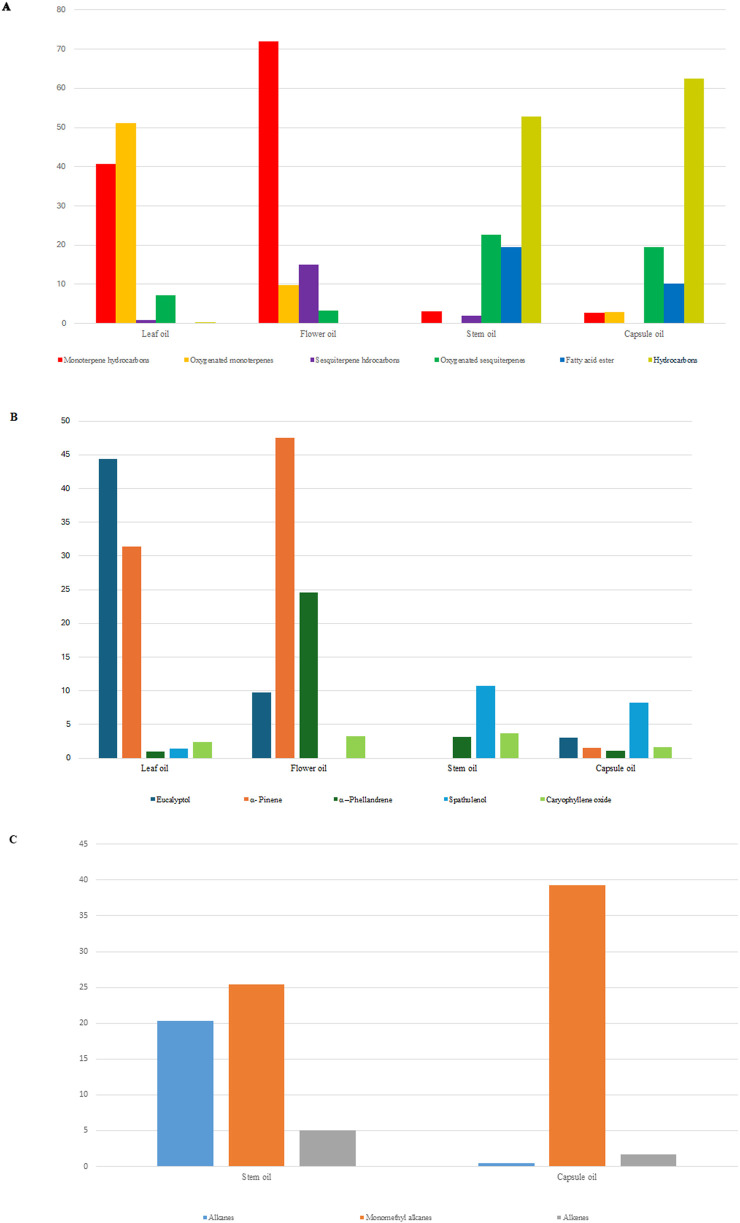
The compositional variation of *C. subulatus* leaf, flower, stem, and capsule oils displays overall classes of compounds **(A)**, the major monoterpenes and sesquiterpenes **(B)**, and the three major hydrocarbons in stem and capsule oils **(C)**.

The components identified in the tested oils can be categorized into many classes, such as terpenes, including monoterpenes and sesquiterpenes; fatty acids; fatty acid esters; and hydrocarbons, including alkanes, alkenes, monomethyl alkanes, alcohols, and ketones, which together represent the qualitative variation. Although the major class in leaf (91.81%) and flower (81.82%) oils was monoterpenes, the aliphatic hydrocarbons are frequently found in stem and fruit oils (Figure 2A). There were only two compounds characterized in all studied organs, the monoterpene hydrocarbon *α*-phellandrene and the oxygenated sesquiterpene caryophyllene oxide.

The most common monoterpene in the genus *Callistemon* is eucalyptol, an oxygenated monoterpene that presents 44.36% of the *C. subulatus* leaf oil, 9.76% of the flower oil, and 2.99% of fruit oil, but it is not found in the stem oil. A monoterpene hydrocarbon, *α*-pinene, comprises 47.52% of flower oil, 31.41% of leaf oil, and 1.51% of fruit oil, and it is also absent from stem essential oil. The oxygenated sesquiterpenes, which are present in significant amounts in *C. subulatus* oil, especially spathulenol, are prevalent in the stem (10.77%) and fruit oils (8.18%). The most predominant terpenes are illustrated in Figure 2B. The most predominant compounds found in fruit (39.25%) and stem (25.46%) oils are monomethyl alkanes (11 compounds), mainly 3-methylalkanes (anteiso) (7 compounds) (Figure 2C). These long-chain 3-methylalkanes accounted for 34.47% of fruit oils, 7.35% of stem oils, and 0.27% of leaf oils.

### 3.2 Effects of the leaf oil of *C. subulatus* on the lung/body weight index

**FIGURE 3:**
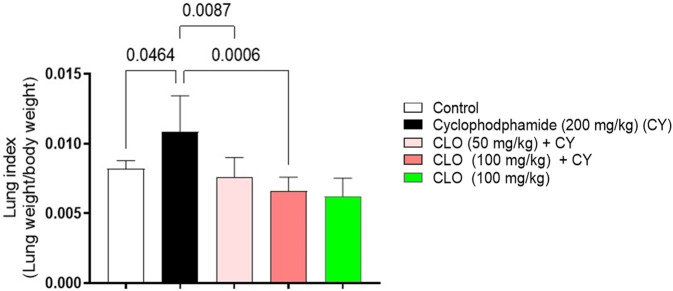
Effect of CLO treatment on the lung index in rats with CY-induced pulmonary toxicity. Data are presented as the mean ± SD (n = 6) at *p* < 0.05 using one-way analysis of variance (ANOVA), followed by Tukey’s post-hoc test.

Cyclophosphamide-treated rats showed a significant 1.32-fold increase in the lung index compared with the control group. However, treatment with either low or high doses of CLO resulted in a significant reduction in the lung index compared to cyclophosphamide-treated rats, by 1.43 and 1.64 times, respectively (Figure 3).

### 3.3 Effects of the leaf oil of *C. subulatus* on oxidative stress markers

**FIGURE 4:**
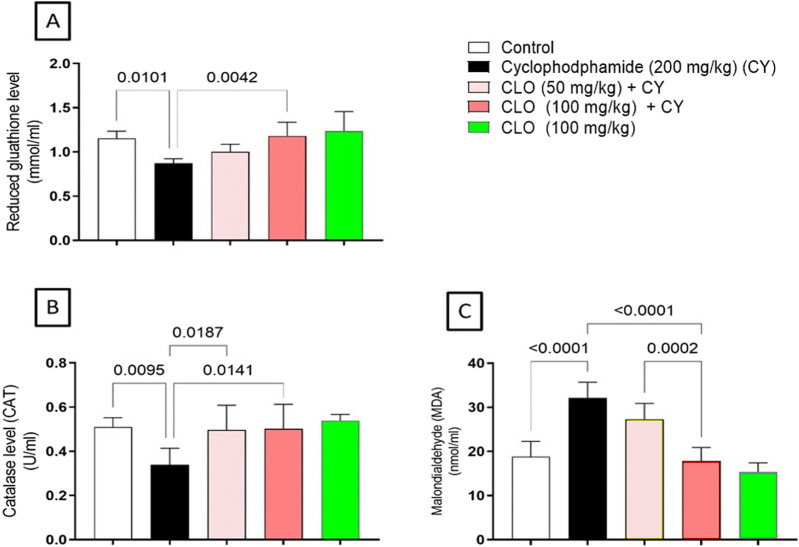
Effect of treatment with CLO on pulmonary GSH **(A)**, CAT **(B)**, and MDA **(C)** levels in an experimental model of pulmonary toxicity induced by CY. Data are presented as the mean ± SD (n = 6) at *p* < 0.05 using ANOVA, followed by Tukey’s post-hoc test.

As shown in Figure 4, a significant decrease in the GSH level was found when comparing the CY-treated group and the control group. On the other hand, treatment with CLO at a dose of 100 mg/kg resulted in a significant increase in GSH levels compared with cyclophosphamide-treated rats (Figure 4A). Additionally, the CAT level was found to be significantly reduced following CY treatment relative to the negative control group. In contrast, treatment with either dose of CLO, 50 mg/kg and 100 mg/kg, illustrated a significant elevation in the GSH level compared to CY-treated rats (Figure 4B). Moreover, the lipid peroxidation level was elevated significantly in CY-treated rats compared to control rats. Interestingly, treatment with CLO at a dose of 100 mg/kg revealed a significant reduction in the MDA level compared to both CY-treated and low-dose CLO-treated rats (Figure 4C).

### 3.4 Effects of the leaf oil of *C. subulatus* on *α*-smooth muscle actin expression

**FIGURE 5:**
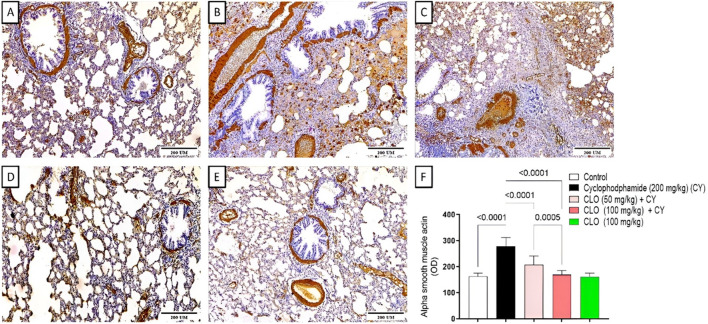
Expression of alpha smooth muscle actin by immunohistochemical staining (×100). Photomicrographs of stained sections for the control group **(A)** CY-treated group (200 mg/kg) **(B)** CY + CLO-treated group (50 mg/kg) **(C)** CY + CLO-treated group (100 mg/kg) **(D)** and CLO-treated group (100 mg/kg) **(E)**. Results are quantified as OD of the stained regions **(F)**. Brown color (positive) indicates specific immunostaining of alpha smooth muscle actin, and blue color (negative) indicates hematoxylin staining (n = 3). Data are presented as the means + S.D. across 15 different sections at p < 0.05.

To determine the level of lung fibrosis in different groups, the expression of *α*-SMA was estimated using immunohistochemical staining. The immunohistochemical staining was quantified as optical density (OD) of the stained regions using image analysis software, and the results are represented in Figure 5. Cyclophosphamide treatment (Figure 5B) showed a significant increase in *α*-SMA expression by 71.09% compared to the control group (Figure 5A), as evident from a significant increase in OD. In contrast, treatment of animals with both doses of CLO at a dose of 50 mg/kg (Figure 5C) and 100 mg/kg (Figure 5D) markedly decreased *α*-SMA expression relative to the CY-treated group by 34.39% and 64.91%, respectively. An insignificant change in *α*-SMA expression was observed in the group treated with CLO alone (Figure 5E).

### 3.5 Histopathological examination of lung tissues

**FIGURE 6:**
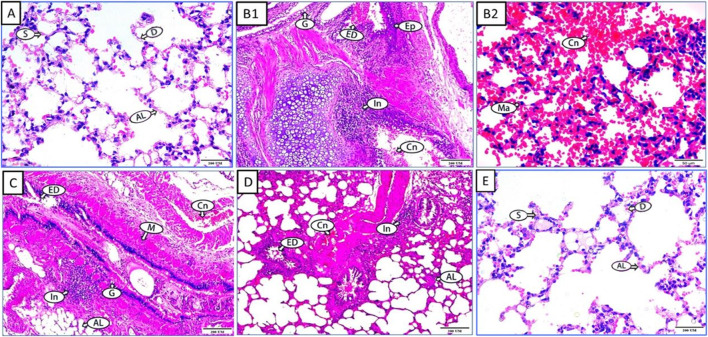
Effect of CLO treatment on CY-induced histological alterations of rat pulmonary tissues. Photomicrographs of hematoxylin and eosin-stained sections from the control group **(A)** CY-treated group (200 mg/kg) **(B1,B2)** CY + CLO-treated group (50 mg/kg) **(C)** CY + CLO-treated group (100 mg/kg) **(D)** and CLO-treated group (100 mg/kg) **(E)** with ×100 magnification power. Interalveolar septum (S), alveolar duct (D), alveolar wall thickness (AL), muscle thickness (M), inflammatory cells (In), congested blood vessels (Cn), epithelial desquamation (ED), epithelium hyperplasia (EP), and goblet cells (G).

Histological examination displayed changes in lung tissue structure among the examined groups (Figure 6). Sections from the negative control group presented the normal structure of alveoli (AL), interalveolar septum (S), and alveolar duct (D) inside lung tissue (Figure 6A). Lung sections from the CY-treated group highlighted severe lung degeneration, evidenced by bronchus-exhibited epithelium with hyperplasia (EP), desquamation (ED), and an increased number of goblet cells (G). Inflammation (In) and congestion (Cn) in the submucosal layer were also observed (Figure 6B1). In addition, the higher-power section between the alveoli disclosed congestion (Cn) and inflammatory cells, including macrophages (Ma) (Figure 6B2). Lung sections from rats treated with CLO (50 mg/kg) exhibited some epithelial desquamation (ED) with a normal number of goblet cells (G), a moderate increase in alveolar wall (AL) along with muscle thickness (M), aggregated inflammatory cells (In), and congested blood vessels (Cn) (Figure 6C). Moreover, CLO (100 mg/kg) improved lung tissue architecture, showing reduced epithelial desquamation (ED), inflammatory cell aggregation (In), congested blood vessels (Cn), and thickened alveoli walls (AL) (Figure 6D). The CLO alone-treated group represented the same records as the negative control group, with regular alveolar duct (D) and alveoli (AL) composition (Figure 6E).

**FIGURE 7:**
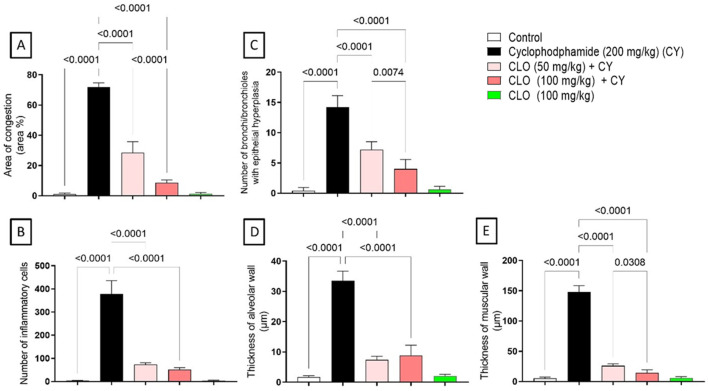
Quantitative scoring of histological changes following CLO and CY treatment in terms of percentage area of blood vessel congestion **(A)**, number of inflammatory cells **(B)**, number of bronchioles with epithelial hyperplasia **(C)**, alveolar wall thickness **(D)**, and muscular wall thickness **(E)**. The data are presented as the means ± S.D. (n = 5) and analyzed using one-way ANOVA, followed by Tukey’s *post hoc* test, at *p* < 0.05.

Quantitative scoring of the area percentage of congestion in the lung tissue (Figure 7A) showed a significant increase in CY-treated rats compared to the control group. On the other hand, treatment with CLO, at either 50 or 100 mg/kg, resulted in a significant decrease in the percentage area of congestion compared with the CY-treated group. It is worth noting that a group of rats treated with CLO (100 mg/kg) exhibited a significant reduction compared to a lower dose (50 mg/kg). Quantitative scoring of the number of inflammatory cells shown in pulmonary tissue (Figure 7B) showed an increase in the group treated with CY compared to the control. However, CLO in 50 and 100 mg/kg achieved a significant reduction compared to the CY-treated group. In addition, the number of bronchioles and bronchi with epithelial hyperplasia was significantly increased in the CY-treated group relative to the control group. In contrast, the groups treated with CLO 50 or 100 mg/kg showed significant amelioration in the number of hyperplastic bronchioles compared to CY-treated rats (Figure 7C). Finally, for the thickness of the alveolar wall (Figure 7D) and the thickness of the muscular wall (Figure 7E), the quantitative scoring showed an increase in CY-administered rats compared to the corresponding control group. Co-treatment with CLO, at either 50 or 100 mg/kg, resulted in a significant reduction in the thickness of the alveolar and muscular walls compared with CY-treated rats. Moreover, the higher dose of CLO (100 mg/kg) achieved a significant decrease in the thickness of the muscular wall compared to the lower oil dose (50 mg/kg).

### 3.6 Molecular docking study

Molecular docking was conducted to identify the possible mechanism of action of the major compounds eucalyptol and *α*-pinene. The human CAT and the TGF-*β* receptor were selected as potential targets. The TGF-*β* receptor increases the transcription of the profibrotic *α*-SMA; accordingly, it was selected as a potential target.

**FIGURE 8:**
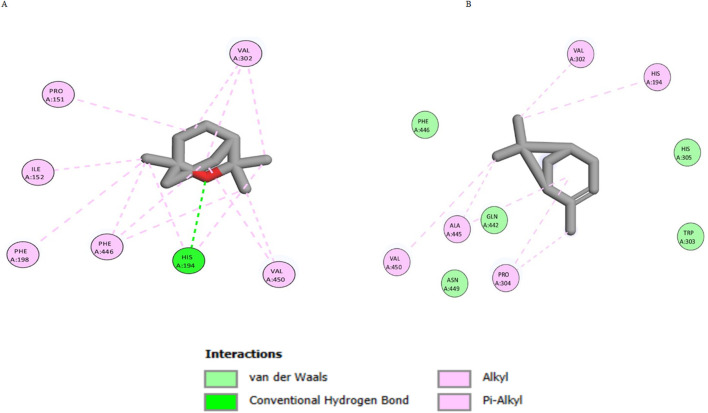
2D interaction diagram of eucalyptol **(A)** and *α*-pinene **(B)** with human CAT, PDB ID: 8hid.

For the docking of human CAT, both eucalyptol and *α*-pinene achieved excellent docking scores of −7.9 and −6.8 kcal/mol, respectively. As shown in Figure 8, eucalyptol was able to interact with CAT residues through hydrogen bond interactions with His194, in addition to several hydrophobic interactions with Pro151, Ile152, Phe198, Val302, Phe446, and Val450. Similarly, *α*-pinene interacted with CAT residues through several hydrophobic interactions with His194, Val302, Pro304, Ala445, and Val450.

**FIGURE 9:**
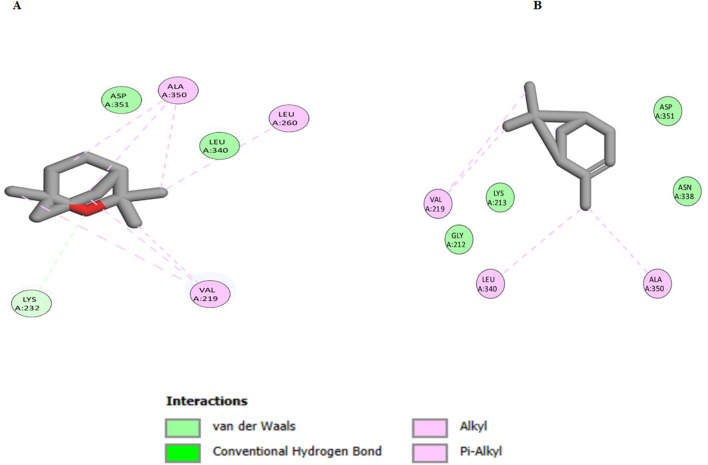
2D interaction diagram of eucalyptol **(A)** and *α*-pinene **(B)** with the TGF-*β* receptor, PDB ID: 6bby.

For the docking of the TGF-*β* receptor, eucalyptol achieved a docking score of −7.4 kcal/mol, while *α*-pinene achieved −6.8 kcal/mol. As shown in Figure 9, eucalyptol interacted with Val219, Lys232, Leu260, and Ala350 through hydrophobic interactions. In a similar manner, *α*-pinene interacted with Val219, Leu340, and Ala350 through hydrophobic interactions.

## 4 Discussion

The essential oils of *C. subulatus* leaf, flower, stem, and fruit were detected using GC/MS, and it was revealed that the leaf oil gave the highest yield. Each oil contains a different number of compounds, indicating a qualitative variation between the oils, ranging from a few terpenes in flower oil to 25 compounds in the fruit oil. The results are somewhat consistent with the earlier study on Egyptian *C. subulatus*, which found that the two main components of leaf oil are eucalyptol (48.97%) and *α*-pinene (31.59%), while *α*-terpineol (7.84%) occupies the third position, followed by limonene (5.27%). It also confirms that the primary monoterpene in flower oil is *α*-pinene (82.64%), while the second and third are eucalyptol (11.45%) and *α*-phellandrene (3.84%) (Ibrahim and Moussa, 2020). However, another study revealed that the major monoterpene in the leaf oil of *C. subulatus* was *α*-pinene (47.06%), followed by eucalyptol (16.13%) and *α*-terpineol (15.27%) (Ibrahim et al., 2020). The differences in the type and percentage of major components of the oils are attributed to variations in environmental conditions, collection season, and analytical methods (El-Nashar et al., 2021; Elhawary et al., 2021; El-Nashar et al., 2024).

Cyclophosphamide has been shown to cause pulmonary toxicity, which can manifest as either early-onset interstitial pneumonia or late-onset pulmonary fibrosis (Voelcker, 2020). The CY metabolites enhance the conversion of oxygen to ROS, which recruit inflammatory cells to the site of injury and cause a buildup of fluid known as pulmonary edema (Barberino et al., 2023; Zengin et al., 2023; Abdallah et al., 2022). The lungs of rats administered CY frequently exhibited this edema, a common sign of CY-induced pneumonitis, which is reflected by an increased in lung/body weight ratio. In our study, CY increased the lung index by 1.32-fold compared with control rats. This is consistent with a previous study in which two doses of CY, 50 and 75 mg/kg, increased the lung index by 1.46- and 2.02-fold, respectively, compared with normal mouse lungs (Said et al., 2016).

When ROS accumulates, oxidative stress is induced, which lowers the antioxidant contents of GSH and CAT in the systemic defense and increases the level of pulmonary MDA, a byproduct of lipid peroxidation (Mohammed et al., 2024). Our findings demonstrated that CY-treated rats exhibited lower lung GSH and CAT levels and higher MDA levels compared with the control group. These findings are consistent with previous research that has shown the impact of CY on pulmonary GSH, CAT, and MDA levels (Saghir et al., 2020; Habibi et al., 2020; Shokrzadeh et al., 2014b; Bhattacharjee et al., 2015).

CLO was co-administered with CY to the rats in an attempt to mitigate the lung-damaging effects of the CY because CLO contains eucalyptol (44.34%), which has been reported to possess mucolytic, cytotoxic, antioxidant, and anti-inflammatory properties (Chandorkar et al., 2021). It also demonstrated effective therapeutic uses in inflammatory pulmonary diseases such as asthma, bronchitis, and COPD.

In lung tissues, eucalyptol (10 mg/mL) decreased the elevated pulmonary MDA level induced by cigarette smoking (Kennedy-Feitosa et al., 2019). Furthermore, eucalyptol (50 µM) pre-treatment increased *in vitro* GSH and CAT levels, which were decreased by the cigarette extract on the human bronchial epithelial cell line (BEAS-2B) (Reis et al., 2021).

In addition to eucalyptol, CLO also contains *α*-pinene (31.41%), which demonstrated potent antioxidant properties, the ability to scavenge DPPH-free radicals at 12.57 mg/mL, and a high reducing power of 213.7 μg/mL in converting the ferricyanide complex to the ferrous form (Wang et al., 2019). This terpene also showed apoptotic and anti-metastatic activities in experimental metastatic melanoma, decreasing the number of lung tumor nodules (Salehi et al., 2019).

As a result, our findings regarding the antioxidant capacity of CLO (50 and 100 mg/kg) to prevent GSH and CAT depletion and reduce MDA levels in the lung tissue were ascribed to the antioxidant-rich content of eucalyptol and *α*-pinene. Additionally, a recent study found that the *C. subulatus* leaf extract was able to restore the GSH, CAT, and MDA levels that the CY had disrupted in rat brain tissue (Rabie et al., 2023).

Pulmonary fibrosis is characterized by the accumulation of myofibroblasts, the activated form of fibroblasts, which are responsible for the pathogenic synthesis of collagen and the deposition of the extracellular matrix in the alveolar structures (Mostafa et al., 2022).

Increased expression of *α*-SMA, a key marker of myofibroblasts, indicates fibrosis, as reported in CY-induced pneumotoxicity in the literature (Chen et al., 2021). Our results from the immunohistochemical staining likewise showed this elevation; CY increased the expression of the fibrotic signal *α*-SMA by 71.09% compared to the control group. In contrast, rats co-treated with CLO at 50 and 100 mg/kg showed a decrease in *α*-SMA by 34.39% and 64.91%, respectively, compared to the CY-treated group.

As evidenced by a decrease in the level of *α*-SMA, CLO was able to reduce the development of fibroblasts into myofibroblasts. This possibility could be explained by the fact that CLO contains more than 40% eucalyptol as investigations have shown that eucalyptol can improve lung tissues that are injured and fibrotic (Dey et al., 2022). Eucalyptol significantly reduced lung inflammation, macrophage polarization, and myofibroblast activation, hence reducing the expression of *α*-SMA (Rui et al., 2022).

Another study revealed that co-treating eucalyptol and CY allowed the GSH levels in the liver and heart tissue to return to normal levels (Abdallah et al., 2019). These reports suggest that eucalyptol-containing plants could be used as a co-drug to treat CY-induced lung fibrosis.

Cyclophosphamide recruits inflammatory cells, such as macrophages, whose activation leads to an increase in the expression of the pro-fibrotic cytokine TGF-*β* (El-Kholy et al., 2017). The TGF-*β* signaling pathway regulates the development of fibrosis by upregulating the transcription of profibrotic *α*-SMA.

The purpose of the docking study was to determine the predictive mechanism of action of the main *C. subulatus* oil constituents, eucalyptol and *α*-pinene, on human CAT and TGF-*β* receptors. The two investigated monoterpenes exhibited favorable docking scores by interacting with active sites through hydrogen bonding and hydrophobic interactions. Eucalyptol exhibits the greatest results, with lower binding energies and good binding affinities to both targets: CAT and TGF-*β*.

Patients with CY-induced pulmonary fibrosis exhibited atypical alveolar epithelial cells, hyperplasia of type II pneumocytes, interstitial edema, proliferation of activated fibroblasts, and fibrosis in the histopathological images of their lung tissue (Bartl et al., 2023).

According to our histopathological results, the lung tissues from the control group of mice exhibited a normal alveolar lining, a normal alveolar duct, and a normal inter-alveolar septum, . However, the histology of the group that received CY treatment revealed severe lung tissue damage and the absence of the normal alveolar architecture. It demonstrated bronchial epithelial hyperplasia, congestion, desquamation, and inflammation in addition to an increase in goblet cells, inflammatory cells, and macrophages. These CY-induced alterations were previously reported in the lung tissues of mice at various intraperitoneal dosages of CY. It caused noticeable thickening and congestion of the interalveolar septa, desquamation of the intrabronchiolar epithelium, alveolar edema with congested blood vessels, infiltration of inflammatory cells, primarily macrophages and lymphocytes, and an increase in goblet cells (Abd El‐Ghafar et al., 2021; Akl et al., 2022).

Except for minor epithelial desquamation and mild inflammatory cell infiltration, CLO could considerably prevent lung tissue deterioration, protect against pulmonary injury, and maintain a normal number of goblet cells. CLO (100 mg/kg) improved lung tissue architecture more effectively than the lower dose.

Eucalyptol (50 mg/kg), which demonstrated a considerable reduction in inflammatory cell accumulation and collagen deposition in lung tissues of mice exhibiting pulmonary fibrosis, may be responsible for this ameliorating impact of CLO. Additionally, eucalyptol, at a dose of 10 mg/mL, promoted the formation of normal alveoli and thin alveolar septa,, mitigating the effects of cigarette smoking on lung tissue, including alveolar enlargement and septal disruption (Kennedy-Feitosa et al., 2019; Rui et al., 2022).

To ensure the safety of the volatile oil derived from Egyptian *C. subulatus* leaves, preliminary research revealed that the IC_50_ value of 771.04 μg/mL indicated no cytotoxic effect against the human lung fibroblast WI-38 cell line (Ibrahim and Moussa, 2020). On the other hand, the essential oils of *C. citrinus* flowers and leaves (100 μg/mL) showed cytotoxicity against human lung cancer (A549) cell lines with IC_50_ values of 77.8 and 84 μg/mL, respectively (Kumar et al., 2015). According to findings from a preclinical animal model, patients with lung cancer on CY regimens or pneumonitis may benefit from using natural, safe essential oils from the aromatic genus *Callistemon.* However, pharmacokinetic, safety, and clinical validation are required to validate the application. *Callistemon* species with eucalyptol as the primary terpene are preferred for application due to their lower binding energy for CAT and TGF-*β* receptors, according to an *in silico* study. Further experimental validation, such as enzyme activity assays and signaling pathway studies, could provide a clearer explanation of the proposed pathways.

## 5 Conclusion

In this study, we demonstrated chemical variation in the oils of different organs of *C. subulatus*, with eucalyptol being the major component of CLO, which exhibited the highest yield and was selected for the biological study. Both doses of CLO (50 and 100 mg/kg) could mitigate pneumotoxicity caused by CY (200 mg/kg) in the lung tissue of mice. According to our findings, CY altered the oxidative balance, as evidenced by lower GSH and CAT levels and an increased MDA level. However, CLO was able to restore the cellular redox status and lower lipid peroxidation. Additionally, CY promotes the differentiation of fibroblasts into myofibroblasts, resulting in the overexpression of *α*-SMA. Furthermore, both CLO doses counteracted these effects and downregulated *α*-SMA. Finally, CLO prevented lung tissue damage and restored the destruction caused by CY, reducing epithelial desquamation and relieving congestion and inflammation. The study indicated that the leaf oil of *C. subulatus* could protect rat lungs from the damaging effects of CY and that it had no adverse effects on the general health of the rats. As a result, this oil may be a promising natural candidate for protecting the lungs from CY-induced pneumotoxicity. To maximize the therapeutic benefit of CLO, the exact dosage, delivery method, and pharmacokinetic studies should be carefully considered and studied through clinical trials.

## Data Availability

The original contributions presented in the study are included in the article/supplementary material, further inquiries can be directed to the corresponding authors.
